# Pharmacological strategies to reduce exacerbation risk in COPD: a narrative review

**DOI:** 10.1186/s12931-016-0425-5

**Published:** 2016-09-10

**Authors:** Marc Miravitlles, Anthony D’Urzo, Dave Singh, Vladimir Koblizek

**Affiliations:** 1 Pneumology Department, Hospital General Universitari Vall d’Hebron, Pg. Vall d’Hebron 119-129, 08035 Barcelona, Spain; 2 Department of Family and Community Medicine, University of Toronto, 1670 Dufferin Street, Suite 107, Toronto, ON M6H 3M2 Canada; 3 University of Manchester, Medicines Evaluation Unit, University Hospital of South Manchester Foundation Trust, Southmoor Road, Manchester, M23 9QZ UK; 4 Department of Pneumology, Charles University in Prague, Faculty of Medicine in Hradec Kralove, Simkova 870, Hradec Kralove 1, 500 38 Czech Republic

**Keywords:** Emphysema, Bronchitis, Exacerbation, Risk factors, Guidelines, Prevention, Treatment, Phenotypes

## Abstract

Identifying patients at risk of exacerbations and managing them appropriately to reduce this risk represents an important clinical challenge. Numerous treatments have been assessed for the prevention of exacerbations and their efficacy may differ by patient phenotype. Given their centrality in the treatment of COPD, there is strong rationale for maximizing bronchodilation as an initial strategy to reduce exacerbation risk irrespective of patient phenotype. Therefore, in patients assessed as frequent exacerbators (>1 exacerbation/year) we propose initial bronchodilator treatment with a long-acting muscarinic antagonist (LAMA)/ long-acting β_2_-agonist (LABA). For those patients who continue to experience >1 exacerbation/year despite maximal bronchodilation, we advocate treating according to patient phenotype. Based on currently available data on adding inhaled corticosteroids (ICS) to a LABA, ICS might be added to a LABA/LAMA combination in exacerbating patients who have an asthma-COPD overlap syndrome or high blood eosinophil counts, while in exacerbators with chronic bronchitis, consideration should be given to treating with a phosphodiesterase (PDE)-4 inhibitor (roflumilast) or high-dose mucolytic agents. For those patients who experience frequent bacterial exacerbations and/or bronchiectasis, addition of mucolytic agents or a macrolide antibiotic (e.g. azithromycin) should be considered. In all patients at risk of exacerbations, pulmonary rehabilitation should be included as part of a comprehensive management plan.

**Electronic supplementary material:**

The online version of this article (doi:10.1186/s12931-016-0425-5) contains supplementary material, which is available to authorized users.

## Background

The consequences of a chronic obstructive pulmonary disease (COPD) exacerbation are many. Adverse effects of an exacerbation include a negative impact on patient quality of life [1, 2], an effect on symptoms and lung function that are associated with a lengthy recovery [3], an accelerated rate of decline in lung function [4, 5] and hospital admissions [6]. Moreover, exacerbations may result in significant mortality, particularly in cases that require hospitalization [7] and are associated with a high socioeconomic burden, with exacerbations accounting for most of total COPD healthcare expenditure [8]. Therefore, any COPD exacerbation should not be underestimated given their detrimental impact, and their prevention is a key goal of COPD treatment [9]. It is useful to consider that exacerbations are heterogeneous events and the nature of exacerbations is likely to differ between different subgroups of patients with COPD, such as patients with chronic bronchitis or severe emphysema [10].

This narrative review focuses on identifying and managing patients with COPD according to their risk and type of exacerbation and discusses current and evolving preventative strategies. Selection of the studies included in the ‘Pharmacologic therapies to prevent COPD exacerbations’ section of this paper were identified via a PubMed search [details provided in Additional file 1]. Given the nature of this review, the studies referred to herein did not need to adhere to any pre-specified exacerbation definition.

### Identifying patients at risk of COPD exacerbations

#### How do we define an exacerbation of COPD?

Although most guidelines define exacerbations similarly, there are differences in the exact definition used [see Additional file 1: Table S1, which provides a non-comprehensive list of exacerbation definitions used within a selection of societal guideline documents]. Variations in the definition of exacerbations within clinical studies also exist [11]. Clinical trials use event-based definitions of exacerbation, which necessitate an increase in symptoms and the use of healthcare resources [12, 13], and grade exacerbations based on their severity, e.g. moderate exacerbations as those requiring treatment with systemic corticosteroids/antibiotics and severe exacerbations as those necessitating hospitalization [14–16]. In an attempt to establish a uniform definition of exacerbations to be used as an outcome measure in clinical trials, the European Respiratory Society (ERS) and the American Thoracic Society (ATS) task force defined an exacerbation as an increase in respiratory symptoms over baseline that usually requires a change in therapy [17]. It is relevant to note that many exacerbations go unreported and untreated [18, 19].

#### Key risk factors for COPD exacerbation

Numerous factors may contribute to a COPD exacerbation, the most frequent cause of which is a respiratory tract infection [20–22]. However, other factors also play a contributing role, as shown in Table 1, including a change in weather/temperature [23], air pollution [24], and comorbid conditions [6, 25].

**Table 1:** Risk factors for exacerbations of chronic obstructive pulmonary disease [6, 21–23, 25, 122]

Risk factors	
•	Older age
•	Lower body mass index
•	Continued smoking
•	Poor exercise capacity
•	Severity of airflow obstruction
•	Previous exacerbations (including hospitalizations for exacerbations)
•	Longer duration of chronic obstructive pulmonary disease
•	Co-morbidities (e.g. bronchiectasis, cardiovascular disease, pulmonary hypertension, gastroesophageal reflux)
•	Respiratory tract infections (viral and bacterial
•	Chronic bronchial mucus production
•	Environmental pollutants
•	Colder weather/temperature

Some patients may be more susceptible to developing COPD exacerbations than others, and are generally referred to as ‘frequent exacerbators’. In the Evaluation of COPD Longitudinally to Identify Predictive Surrogate Endpoints (ECLIPSE) study, an analysis of exacerbations in 2138 patients demonstrated that the single best predictor of frequent exacerbations was an exacerbation in the preceding year [14]. In addition, exacerbations were more frequent and more severe (associated with hospitalization) in patients with advanced airflow obstruction [14]: 22 % of patients with Global initiative for chronic Obstructive Lung Disease (GOLD) 2 disease, 33 % with GOLD 3 and 47 % with GOLD 4 disease had frequent exacerbations (≥2) in the first year of follow-up. Other factors associated with increased exacerbation frequency were increased rate of lung function decline (100 mL decrease in forced expiratory volume in 1 s (FEV_1_) and a history of reflux or heartburn [14]. In addition, a low level of physical activity can also predict future exacerbations [26, 27], independent of pulmonary function and previous exacerbation history [27].

Several studies have demonstrated that a chronic bronchitis phenotype is associated with an increased risk of exacerbations in patients with COPD [28–30]. However, patients with an emphysematous phenotype are also likely to exacerbate more frequently, in particular if they have severe emphysema [31].

#### Assessment of exacerbation risk: what do guidelines say?

COPD guidelines have historically just used lung function to grade the severity of disease, and guide treatment strategies. A more complex approach has been adopted recently, incorporating other clinical features to define clinical phenotypes. A phenotype is defined as “a single or combination of disease attributes that describe differences between individuals with COPD as they relate to clinically meaningful outcomes (symptoms, exacerbations, response to therapy, rate of disease progression, or death)” [32]. The aim of phenotyping is to enable identification of patient groups that have distinctive prognostic or therapeutic characteristics, thereby enabling a personalized approach to treatment.

The definition of the “frequent exacerbator” phenotype, based on findings from the ECLIPSE study [14], has been incorporated into many COPD guidelines. Current GOLD strategy advocates assessment of exacerbation risk by: (i) GOLD spirometric classification, with GOLD 3 and 4 indicating high risk; or (ii) a history of exacerbations, with ≥2 exacerbations (or ≥1 exacerbation leading to hospitalization) in the previous year indicating high risk [9]. GOLD subdivides patients into four categories, A, B, C or D, on the basis of symptoms and risk, with the aim of using this clinical assessment to make treatment decisions. Guidelines differ in how the risk of future exacerbations is assessed (see Additional file 1: Table S2), with a previous history of exacerbations being a common feature of many [9, 33–38], but other characteristics such as FEV_1_ included in some [9, 34, 35, 37], but not all, guidelines, e.g. Spanish GesEPOC and the Saudi Thoracic Society [33, 36, 38]. In fact, the Spanish (GesEPOC) guidelines have adopted a different approach to other national guidelines. Four clinical phenotypes are defined [33]: non-exacerbators (<2 exacerbations/year), an asthma-COPD overlap syndrome phenotype (ACOS), and two groups of frequent exacerbators (≥2 exacerbations/year), specifically exacerbators with emphysema and exacerbators with chronic bronchitis. Importantly, patients with ACOS experience more frequent and severe exacerbations, as well as worse disease-related quality of life, compared with patients having COPD [33, 39–41].

The main problem associated with the implementation of the GOLD strategy comes from the inclusion of patients with either poor lung function or exacerbations or both into the same categories C or D. An analysis of several large study databases has reported that, of those patients categorized as GOLD D (i.e. at high risk), most (63–79 %) qualify based solely on airflow limitation (FEV_1_ < 50 % predicted) and not prior exacerbation frequency [42]. This heterogeneity of patients qualifying by different criteria (airflow restriction or exacerbation frequency, or both) for GOLD groups C and D [9] can prove a challenge for physicians wishing to individualize treatment, as patients in these categories may have different levels of exacerbation risk and may require different treatment approaches. For example, the COPDGene study showed significant (*p* < 0.0001) differences in exacerbation rates depending on whether patients were categorized as GOLD D based on lung function alone (0.89 exacerbations/person-year), on previous exacerbation history only (1.34 exacerbations/person-year) or both criteria (1.86 exacerbations/person-year) [43].

#### Phenotypes of COPD exacerbations

Bafadhel et al have demonstrated that phenotypes of exacerbations exist comprising four biologic exacerbation clusters: bacterial, viral, eosinophilic and pauci-inflammatory [10]. The type of inflammatory response during an exacerbation (eosinophilic vs neutrophilic) may depend on the patient phenotype in stable state [44–46]. Individuals with eosinophilic exacerbations tend to have increased levels of sputum eosinophils in a stable condition, suggesting that patient phenotypes in stable COPD and exacerbations of COPD are closely related [10, 46]. Eosinophilic airway inflammation occurs in exacerbations of both COPD and asthma [47], and it has previously been shown that peripheral and sputum eosinophil counts are higher in patients with concomitant COPD and asthma, i.e. ACOS compared with COPD alone [48, 49]. Montuschi et al describe the possibility of an association between (1) infective exacerbations and a chronic bronchitis phenotype; (2) pauci-inflammatory exacerbations with an emphysema phenotype; and (3) eosinophilic exacerbations with an ACOS phenotype [50]. We agree with the authors that confirmatory studies are required in this regard, particularly in light of data showing increased sputum neutrophil counts in individuals with ACOS [51]. At present, therefore, sputum eosinophilia or neutrophilia cannot be used as a distinguishing feature for ACOS [52].

Phenotypes of COPD exacerbations is an interesting area of discussion and deserves greater focus than can be given in the current paper. Nevertheless, in addition to demonstrating specific biologic exacerbation clusters, Bafadhel et al identified specific biomarkers for clinical phenotypes during exacerbations, i.e. those associated with bacteria (sputum IL-1β), virus (serum CXCL10) and sputum eosinophilia (peripheral eosinophils), which may help direct pharmacological treatment [10].

### Non-pharmacologic therapy to prevent COPD exacerbations

A number of non-pharmacologic strategies exist to prevent COPD exacerbations including smoking cessation, which is perhaps one of the most efficacious interventions in individuals who continue to smoke [53], vaccination, pulmonary rehabilitation and disease management programs/patient education [6].

Pulmonary rehabilitation, defined by the ATS/ERS as a comprehensive intervention that includes thorough patient assessment followed by patient-tailored therapies such as exercise training, education and behaviour change [54], also has convincing evidence of its efficacy in improving quality of life, exercise capacity ond reducing the readmission rates during the period following an exacerbation, although its effect on reducing exacerbation rates are limited [6]. In a meta-analysis of nine trials involving 432 patients with COPD, pulmonary rehabilitation was associated with significant reductions compared with conventional community care in relation to hospital admissions (odds ratio [OR] 0.22, 95 % CI 0.08, 0.58) and mortality (OR 0.28, 95 % CI 0.10, 0.84), with improvements also observed for health-related quality of life (QoL) and exercise capacity [55].

### Pharmacologic therapies to prevent COPD exacerbations

Characterizing COPD phenotypes [56] may identify patients who will respond better to a specific type of treatment and thereby individualize therapy [57, 58]. Unfortunately, clinical trial inclusion criteria do not always match the phenotypes of patients used by guidelines. For example, exacerbation frequency may not be specified as an inclusion criterion, or if exacerbation criteria are specified, the frequency (≥1) may be different than those specified by guidelines (≥2). The following pharmacologic therapies employed in the management of COPD are reviewed with a specific focus on their effect on the risk of exacerbations (Tables 2, 3 and 4), and evidence for use in specific COPD phenotypes.

**Table 2:** Effect of bronchodilators on the risk of COPD exacerbation

Treatment	Annual exacerbation rate	Comparator	Annual exacerbation rate	Reduction in exacerbations	Exacerbation endpoint	Patient population	
						Exacerbation entry criteria (No./year)	FEV_1_ % predicted
LAMA							
Glycopyrronium [69]	0.54	Placebo	0.80	34 %	Secondary (moderate or severe)	NA	≥30– < 80 % (post-BD)
Glycopyrronium [68]	0.43	Placebo	0.59	NS	Secondary (moderate or severe)	NA	≥30– < 80 % (post-BD)
Tiotropium [63]	0.61	Indacaterol	0.79	29 %	Secondary (all)	≥1	≥30– < 50 % (post-BD)
Tiotropium [60]	0.64	Salmeterol	0.72	11 %	Primary (moderate or severe)	≥1	<70 % (post-BD)
Tiotropium [123]	0.69	Placebo	0.87	21 %	Secondary (any)	NS	≤60 % (pre-BD)
Tiotropium [15]	0.73	Placebo	0.85	14 %	Secondary (moderate)	NA	<70 % (post-BD)
Tiotropium [124]	1.17	Placebo	2.46	52 %	Secondary (any)	NA	<80 %
Tiotropium [125]	1.57	Placebo	2.41	35 %	Secondary (any)	≥1	30–65 % (pre-BD)
Tiotropium [126]	0.85	Placebo	1.05	20 %	Secondary (any)	NA	≤60 %
Tiotropium [127]	1.07	Placebo Salmeterol	1.49 1.23	28 % NS	Secondary (any)	NA	≤65 %
Tiotropium [128]	0.73	Placebo	0.96	24 %	Secondary (any)	NA	≤65 %
Tiotropium [129]	0.76	Placebo	0.95	20 %	Secondary (any)	NA	≤65 %
LABA							
Salmeterol [13]	0.97	Placebo	1.13	15 %	Secondary (moderate or severe)	NA	<60 % (pre-BD)
Salmeterol [65]	1.04	Placebo	1.30	20 %	Secondary (any)	≥1	25–70 % (pre-BD)
LAMA/LABA							
Acl/Form [72]	0.42	Placebo	0.29	29 % (pooled data)	Moderate or severe	NA	≥30– < 80 % (post-BD)
IND/GLY [62]	0.84	Glycopyrronium	0.95	12 %	Primary (moderate or severe)	≥1	<50 % (post-BD)
Tiotropium + salmeterol [64]	1.75	Tiotropium	1.61	NS	Secondary (any)	≥1	<65 % (post-BD)
UMEC/VIL [130]	NR	Tiotropium	NR	NS	Secondary endpoint	NA	≤70 % (post-BD)

Studies identified using PubMed search of key terms and limited to clinical trials published in English language and including at least 100 patients. Results were manually filtered for relevance and additional studies added at the author’s discretion. Reductions in exacerbations vs comparator were statistically significant unless otherwise stated (See Additional file 1 for detailed description of term used in searchs)

*Acl* aclidinium, *BD* bronchodilator, *Form* formoterol, *GLY* glycopyrronium, *IND* indacaterol, *LABA* long-acting β_2_-agonist, *LAMA* long-acting muscarinic antagonist, *NA* not applicable, *NR* not reported, *NS* not significant, *P* placebo, *S* salmeterol, *UMEC* umeclidinium, *VIL* vilanterol

**Table 3:** Effect of ICS based therapies on the risk of COPD exacerbation

Treatment [Reference]	Annual exacerbation rate	Comparator	Annual exacerbation rate	Reduction in exacerbations	Exacerbation endpoint	Patient population	
						Exacerbation entry criteria (No./year)	FEV_1_ % predicted
ICS							
FP [131]	0.93	Placebo	0.73	NS	Primary	NA	<90 % (post-BD)
FP [13]	0.93	Placebo	1.13	18 %	Secondary (moderate or severe)	NA	<60 % (pre-BD)
FP [65]	1.05	Placebo	1.30	19 %	Secondary (any)	≥1	25–70 % (pre-BD)
FP [132]	0.99	Placebo	1.32	25 %	Secondary (any)	NA	<85 % (post-BD)
LABA/ICS							
SFC [133]	0.44	Salmeterol	0.48	NS	Primary (severe)	≥1 (last 14 days)	<70 % (pre-BD)
SFC [134]	1.10	Salmeterol	1.59	30.4 %	Primary (moderate or severe)	≥1	<50 %
SFC [13]	0.85	Placebo Salmeterol FP	1.13 0.97 0.93	25 % 12 % 9 %	Secondary (moderate or severe)	NA	<60 % (pre-BD)
SFC [65]	0.97	Placebo	1.30	25 %	Secondary (moderate)	≥1	25–70 % (pre-BD)
SFC [135]	1.06	Salmeterol	1.53	30.5 %	Primary	≥1	<50 %
SFC [61, 136]	1.28	Tiotropium	1.32	NS	Primary (moderate or severe)	NA	<50 % (post-BD)
SFC [137]	0.92	Salmeterol	1.4	35 %	Primary (moderate or severe)	≥2	<50 % (post-BD)
Bud/Form [80]	1.38	Placebo Formeterol Budesonide	1.80 1.85 1.60	23.6 % NS NS	Primary (all)	≥1	<50 % (pre-BD)
Bud/Form [81]	1.42	Placebo Formoterol Budesonide	1.87 1.84 1.59	24 % 23 % NS	Primary (severe)	≥1	<50 %
Bud/Form [138]	0.70 (320/9 μg) 0.79 (160/9 μg)	Formoterol	1.07	34.6 % 25.9 %	Primary (moderate or severe)	≥1	≤50 % (pre-BD)
Bud/Form [139]	NR	Formoterol	NR	36 %	Secondary (any)	≥1	≤50 % (pre-BD)
FF/VI [79]	0.81	Vilanterol	1.11	30 %(Pooled data)	Primary (moderate or severe)	≥1	<70 % (post-BD)
BDP/FOR [140]	0.80	Formoterol	1.12	28 %	Primary (moderate or severe)	≥1	<50 %
BDP/FOR [141]	0.41	Bud/Form Formoterol	0.42 0.43	NS	Primary	≥1	30–50 % (post-BD)
Triple therapy							
Tiotropium + salmeterol + FP [64]	1.37	Tiotropium	1.61	NS	Secondary (any)	≥1	<65 % (post-BD)

Studies identified using PubMed search of key terms and limited to clinical trials published in English language and including at least 100 patients. Results were manually filtered for relevance and additional studies added at the author’s discretion. Reductions in exacerbations vs comparator were statistically significant unless otherwise stated (See Additional file 1 for detailed description of term used in searchs)

*BD* bronchodilator, *BDP/FOR* beclomethasone dipropionate/formoterol fumarate, *FP* fluticasone propionate, *ICS* inhaled corticosteroid, *IND/GLY* indacaterol/glycopyrronium, *LABA* long-acting β_2_-agonist, *SFC* salmeterol/fluticasone propionate, *Bud/Form* budesonide formoterol, *FF/VI* fluticasone furoate/vilanterol, *NA* not applicable, *NR* not reported, *NS* not significant

**Table 4:** Effect of other therapies on the risk of COPD exacerbation

Treatment [Reference]	Annual exacerbation rate	Comparator	Annual exacerbation rate	Reduction in exacerbations	Exacerbation endpoint	Patient population	
						Exacerbation entry criteria (No./year)	FEV_1_ % predicted
Macrolide antibiotics							
Azithromycin [107]	1.94	Placebo	3.22	42 %	Primary (any)	≥3	NA
Azithromycin [108]	1.48	Placebo	1.83	27 %	Primary (moderate or severe)	≥1 severe	<70 % (post-BD)
Azithromycin [142]	2.8	Baseline	6.8	59 %	Moderate or severe	≥4	<50 % (post-BD)
Erythromycin [143]	1	Placebo	2	35 %	Primary (moderate or severe)	NA	30–70 %
Mucolytics							
Carbocysteine [144]	1.01	Placebo	1.35	25 %	Primary (any)	≥2 in 2 years	25–79 %
N-acetylcysteine (high dose) [113]	1.16	Placebo	1.49	22 %	Primary (any)	NA	30–70 %
N-acetylcysteine [114]	0.96	Placebo	1.71	43.9 %	Secondary	≥1	Not stated
N-acetylcysteine [131]	1.00	Placebo	0.73	NS	Primary	NA	<90 % (post-BD)
N-acetylcysteine [145, 146]	1.25	Placebo	1.29	NS	Primary	≥2 (previous 2 years)	40–70 % (post-BD)
PDE-4 inhibitor							
Roflumilast [99]	0.81	Placebo	0.93	NS	Primary (moderate or severe)	≥2 (and chronic cough/sputum)	<50 % (post-BD)
Roflumilast [147]	1.14	Placebo	1.37	16.9 %	Primary (moderate or severe)	≥1	≤50 % (post-BD)
Roflumilast [97]	1.14	Placebo	1.37	17 % (2 studies pooled)	Primary (moderate or severe)	≥1 (and chronic cough/sputum)	<50 % (post-BD)
Roflumilast [148]	0.86	Placebo	0.92	NS	Primary (moderate or severe)	NA	<50 % (post-BD)

Studies identified using PubMed search of key terms and limited to clinical trials published in English language and including at least 100 patients. Results were manually filtered for relevance and additional studies added at the author’s discretion. Reductions in exacerbations vs comparator were statistically significant unless otherwise stated (See Additional file 1 for detailed description of term used in searchs)

*BD* bronchodilator, *NA* not applicable, *NS* not significant

#### Long-acting bronchodilators

Bronchodilation with long-acting muscarinic antagonists (LAMAs) and long-acting β_2_-agonists (LABAs), alone or in combination, is a recommended treatment option for most patients with COPD [9, 34, 35, 59]. Long-acting bronchodilators reduce exacerbation risk by improving expiratory airflow when patients are stable, thereby decreasing air trapping that develops during an exacerbation [11].

Several studies have demonstrated the efficacy of long-acting bronchodilators in reducing exacerbation risk in populations of patients with or without a history of exacerbations (Table 2). Some of these studies have specifically recruited patients with a history of ≥1 exacerbation in the previous year, in order to “enrich” the population with patients more likely to exacerbate during the study period [60–64]. Table 2 shows that long-acting bronchodilators reduce exacerbation rates compared with placebo both in these “enriched” populations and in studies where there were no specific inclusion criteria regarding exacerbation history. This demonstrates the broad ability of long-acting bronchodilators to prevent future exacerbations irrespective of previous exacerbation history.

LABA monotherapy with salmeterol has been shown to reduce the annual rate of moderate or severe exacerbations by 15 % compared with placebo (*p* < 0.001) in a population of patients that may or may not have a history of exacerbations [13] and by 20 % compared with placebo (*p* = 0.003) in patients with more than one exacerbation in the prior year [65] (Table 2). Other LABAs seem to be effective at reducing exacerbations, and in a *post-hoc* pooled analysis of 6-month data from three large Phase III trials of indacaterol 150 and 300 μg once daily versus placebo in 2716 patients with moderate-to-severe COPD, exacerbation rates were significantly reduced by about 30 % with both doses of indacaterol (rate ratios: 0.69; 95 % confidence interval [CI] 0.55, 0.87 and 0.71; 95 % CI 0.57, 0.88, respectively; both *p* = 0.002) [66].

The Understanding Potential Long-term Impacts on Function with Tiotropium (UPLIFT) trial in patients with stable COPD demonstrated a 14 % reduction in exacerbations with tiotropium 18 μg once daily versus usual treatment at 4 years’ follow-up (*p* < 0.001) [15]. In a recent systematic review of 22 studies and >23,000 patients with stable COPD, which included UPLIFT, tiotropium was associated with a 22 % reduction in exacerbations versus placebo (OR 0.78; 95 % CI 0.70, 0.87) [67]. Other LAMAs have also demonstrated efficacy on exacerbations. In the GLOW 1 and 2 studies, where ~95 % of patients had an exacerbation history of 0 or 1 at baseline, glycopyrronium significantly reduced the risk of first moderate or severe exacerbation (by 31 %, *p* < 0.05) and the rate of moderate or severe COPD exacerbations (by 34 %, *p* = 0.001) versus placebo [68, 69]. Data on exacerbations with aclidinium are mixed, with one study showing fewer patients experiencing a moderate or severe exacerbation (hazard ratio [HR] 0.7; 95 % CI 0.55, 0.90; *p* = 0.0046) compared with placebo, and another study showing no effect [70], although the overall exacerbation rate was low, which can reduce the ability to detect an effect of treatment. Studies specifically in patients with prior exacerbations suggest that LAMAs may be more effective than LABAs at reducing the risk of exacerbations [60, 63]. For example, in the Prevention Of Exacerbations with Tiotropium in COPD (POET-COPD) study, which included patients having had at least one exacerbation requiring treatment or hospitalization in the previous year, tiotropium significantly reduced the risk of exacerbations by 17 % versus salmeterol (*p* < 0.001) [60]. Genotyping of a subgroup of patients in this study highlighted that polymorphisms of the β_2_-adrenoceptor can affect exacerbation outcomes in response to salmeterol but not tiotropium [71], and this may have contributed to the difference between treatments in exacerbations.

Data are emerging on the benefits of LABA/LAMA combination therapies in reducing the risk of exacerbations versus various comparators. In a pooled analysis of two 6-month randomized trials, aclidinium/formoterol (LABA/LAMA fixed combination) reduced the rate of moderate or severe exacerbations by 29 % compared with placebo (*p* < 0.05) [72]. In the SPARK study of 2224 patients with GOLD stages 3–4 COPD and ≥1 moderate COPD exacerbation in the past year, indacaterol/glycopyrronium (IND/GLY) significantly reduced the rate of moderate-to-severe exacerbations by 12 % (*p* = 0.038) and all exacerbations by 15 % (*p* = 0.0012) compared with glycopyrronium monotherapy. In addition, IND/GLY reduced the risk of all exacerbations vs tiotropium monotherapy (14 %; *p* = 0.0017) and had a trend to a reduction in the risk of moderate-to-severe exacerbations (10 %; *p* = 0.096) [62]. Recently, the LANTERN study of 744 patients with moderate-to-severe COPD and one or no exacerbations in the previous year reported a significant 31 % reduction in the rate of moderate or severe exacerbations (an exploratory endpoint) with IND/GLY compared with salmeterol/fluticasone propionate (SFC) (*p* < 0.05) [73]. The FLAME study has investigated the effect of IND/GLY compared with SFC on exacerbations as the primary outcome in a population of 3362 patients with a history of exacerbations [74], and demonstrated that IND/GLY was more effective than SFC for reducing the rate of all exacerbations by 11 % (*p* = 0.0003) and moderate or severe exacerbations by 17 % (*p* < 0.001) [74]. Moreover, although the annual rate of severe exacerbations did not reach statistical significance between the two treatment arms due to the low number of events, the time to first severe exacerbation was significantly longer with IND/GLY compared with SFC (19 % lower risk, *p* = 0.046).

Much data are available demonstrating the effectiveness of long-acting bronchodilators in terms of exacerbation reduction in patients with COPD, with studies showing rate reductions compared with placebo of up to 20–30 % with LABAs and 34–35 % with LAMAs (Table 2). Interestingly, combining a LABA and LAMA results in a reduction of risk compared with a LAMA alone and, more importantly, a significant reduction of exacerbation risk compared with a LABA/ICS [62, 73, 74].

#### Inhaled corticosteroids and long-acting bronchodilators

Inhaled corticosteroids (ICS) are generally licensed in COPD for use in combination with a LABA for patients with a history of exacerbations in the past year [9]. GOLD recommends that patients with ≥2 exacerbations (or one hospitalization) should be considered for ICS/LABA treatment [9], and this is also echoed in other guidelines [34–37]. The Spanish, the Finnish and Czech guidelines recommend ICS for patients classified as frequent exacerbators or of the ACOS phenotype [33–35].

Although ICS alone have been shown to produce modest reductions in the occurrence of exacerbations [75], their efficacy is enhanced when combined with a LABA, as demonstrated in a Cochrane review and in a Bayesian network meta-analysis [76, 77]. In the Towards a Revolution in COPD Health (TORCH) study^1^, SFC was associated with a 25 % reduction in exacerbation rate versus placebo (*p* < 0.001), a 12 % reduction versus salmeterol (*p* = 0.002) and a 9 % reduction versus fluticasone propionate (*p* = 0.02) [13] (Table 3). Although SFC was more effective than salmeterol monotherapy for reducing the risk of moderate-to-severe exacerbations, there was no significant difference in the risk of severe exacerbations (requiring hospitalization) [13]. Similarly, in a study^2^ of 797 patients with COPD (FEV_1_ < 50 % predicted and at least one exacerbation in the year prior to the study), SFC significantly reduced the annual rate of moderate/severe exacerbations (30.4 %, *p* < 0.001) versus salmeterol [78]. An analysis of pooled data from two studies^2^ in which participants were given fluticasone furoate/vilanterol also noted significantly fewer moderate or severe exacerbations compared with vilanterol alone (rate ratio 0.7; 95 % CI 0.6, 0.8; *p* < 0.0001) [79]. In patients with severe airflow limitation (GOLD stage III or IV) and ≥1 exacerbation in the previous year^1^, a combination of budesonide/formoterol significantly reduced the risk of exacerbations by 28.5, 22.7 and 29.5 % vs placebo, budesonide and formoterol respectively (*p* < 0.05 for all) [80]. In a similar population^1^, budesonide/formoterol has been reported to reduce severe exacerbations by 24 % vs placebo [81].

The use of ICS in COPD is still controversial [82]. Long-term use of ICS in patients with COPD is associated with an increased risk of pneumonia [83], fractures [84] and diabetes [85], among other potential side effects. With questions concerning the long-term safety of ICS and also data showing similar exacerbation rates with ICS/LABA compared with some long-acting bronchodilators [61], there is emerging consensus that withdrawal of corticosteroids may be appropriate in some patient populations. The WISDOM trial, a 12-month, double-blind, active-controlled study of 2485 patients with severe/very severe COPD and a history of exacerbations, showed no increase in the risk of moderate or severe exacerbations in patients who withdrew from ICS therapy but remained on LABA/LAMA compared with those who remained on ICS with LABA/LAMA [86], although there was an initial drop in FEV_1_ of approximately 40 mL. Corticosteroid withdrawal without long-acting bronchodilation in severe COPD patients may produce a different pattern of results. This observation supports a systematic review of other trials of ICS withdrawal in which there was no conclusive evidence that withdrawal of ICS increased exacerbations [87]. Moreover, switching to LABA from LABA/ICS has been shown to occur without loss of efficacy or increase in exacerbations in patients with a low risk of exacerbations in the INSTEAD (Indacaterol: Switching Non-exacerbating Patients with Moderate COPD From Salmeterol/Fluticasone to Indacaterol) and OPTIMO (Real-Life study On the aPpropriaTeness of treatment In MOderate COPD patients) studies [88, 89].

Patients with ACOS may be particularly likely to benefit from ICS therapy because of the predominance of eosinophilic bronchial inflammation associated with this COPD phenotype [90–92]. In fact, it has been demonstrated that a high Th2 signature in COPD correlates with increased airway wall and blood eosinophil counts, and greater response of hyperinflation to ICS in COPD patients with Th2 type of inflammation [92].

The response to ICS in patients with respiratory disease can be predicted by sputum eosinophil counts [93, 94]. A randomized crossover trial in patients with COPD in the absence of clinical diagnosis of asthma demonstrated an improvement in post-bronchodilator FEV_1_ with ICS treatment (mometasone furoate) compared with placebo in those patients with the greatest degree of sputum eosinophilia [93]. More recently, two post-hoc analyses have shown that blood eosinophil counts may predict the effects of ICS/LABA combination treatment on exacerbation rates [95, 96]. Thus, the identification of patients with eosinophilic inflammation in COPD, even in the absence of asthma, may be a useful phenotype to target those most likely to benefit from ICS therapy, although this approach should be validated in prospective studies first.

There is little doubt from the studies reviewed that an ICS combined with a LABA is an effective intervention for patients with a history of exacerbations. As shown, such treatment is associated with reductions in exacerbations averaging 25 % compared with placebo and between 23–36 % (12 % if we include the TORCH study) compared with LABA monotherapy (Table 3). However, there is growing evidence indicating that not all patients with COPD respond to ICS treatment. Given the potential for pneumonia and other important side effects with ICS in COPD populations, emerging data reveal that it may be possible to withdraw the ICS component in certain patient groups provided that adequate bronchodilation is in place [86–89].

#### Phosphodiesterase-4 inhibitors

PDE-4 inhibitors represent an anti-inflammatory approach that is recognized as a treatment option for patients with COPD who are at high risk of exacerbations and have a chronic bronchitis phenotype [9]. In a pooled analysis of two 1-year, placebo-controlled, double-blind, multicenter studies, roflumilast (a PDE-4 inhibitor) was associated with a 17 % reduction in the rate of moderate-to-severe exacerbations compared with placebo in patients with severe COPD, chronic bronchitis and a history of previous exacerbation (*p* < 0.0003) (Table 4) [97]. A subsequent systematic review of 29 trials with PDE-4 inhibitors (15 roflumilast studies, 14 cilomilast studies) has confirmed these observations [98]. More recently, roflumilast was noted to reduce the rate of moderate or severe exacerbations by 13.2 % vs placebo in high-risk patients (severe COPD, symptoms of chronic bronchitis and ≥2 exacerbations in the previous year) receiving LABA/ICS (of which ~70 % were on triple therapy) in the REACT (Roflumilast and Exacerbations in patients receiving Appropriate Combination Therapy) study [99]. Overall, roflumilast is well tolerated with a safety profile consistent with that expected for the PDE-4 inhibitor class [100]. The most common adverse events with roflumilast are gastrointestinal in nature, specifically diarrhea, nausea and weight loss [101]. Psychiatric events (insomnia, anxiety, depression/suicidal behavior) are also more common with roflumilast in clinical trials [102]. However, studies have demonstrated beneficial effects of roflumilast in terms of glycemic parameters [103] and risk of major adverse cardiovascular events [104].

Overall, current clinical trial data indicate that the PDE-4 inhibitor roflumilast is associated with a reduction in the rate of moderate/severe exacerbations of 13–17 % when compared with placebo in a subset of patients that exhibit symptoms of chronic bronchitis and are at a high risk of exacerbations despite optimal inhaled therapy. However, tolerance of roflumilast may be a hurdle for more extensive use in severe COPD.

#### Macrolide antibiotics

Bacterial infections can trigger COPD exacerbations and, consequently, long-term antibiotic use has been considered as a strategy for the prevention of exacerbations. A meta-analysis of six randomized controlled trials on the use of prophylactic macrolide antibiotics has reported a 37 % risk reduction for exacerbations compared with placebo [105]. A systematic review of seven trials covering more than 3000 patients identified a significant effect of continuous antibiotics for reducing the number of patients experiencing an exacerbation (OR 0.55; 95 % CI 0.30, 0.77) [106]. Since these analyses, a small study (*n* = 92) confirmed a 42 % decrease in exacerbation rate with maintenance azithromycin treatment compared with placebo (OR 0.58; 95 % CI 0.42, 0.79; *p* = 0.001) in patients that suffered at least three exacerbations the previous year while on maximal respiratory medications (Table 4) [107]. The continuous use of antibiotics may raise a concern about bacterial resistance, and an increase in respiratory pathogens resistant to macrolides has been identified with this approach in patients with COPD [108]. Long-term use of macrolides has also been linked to hearing loss and gastrointestinal events [106]. Thus, this approach may be best for patients who experience frequent bacterial exacerbations despite optimal treatment with bronchodilators and anti-inflammatory agents [109] and it may be prudent to limit their use to reference centers with adequate follow-up [59]. A post-hoc analysis of patients treated with continuous azithromycin reported that ex-smokers and patients who are older and have milder COPD may have a better treatment response [110].

Based on the evidence reviewed here, macrolide antibiotic therapy may be a beneficial strategy for patients who suffer frequent bacterial exacerbations while on maximal bronchodilator therapy, as demonstrated in clinical trials that show reductions in exacerbations ranging from 27 to 42 % compared with placebo (Table 4). Nevertheless, given the potential for development of bacterial resistance alongside long-term safety concerns, such treatment needs to be targeted to the most appropriate patient and include careful supervision [111].

#### Mucolytics

Mucolytic therapies, such as carbocysteine or N-acetylcysteine, may represent an attractive treatment strategy for frequent exacerbators with chronic bronchitis, and particularly those who may be unable to receive ICS (Table 4). A systematic review of 30 trials has reported an increased likelihood of being exacerbation free with mucolytic therapy compared with patients without mucolytic therapy (OR 1.84; 95 % CI 1.63, 2.07) [112]. However, it should be noted that there are considerable differences in the patient populations and definitions of exacerbations used in these studies; for example, some of these studies were performed in patients with chronic bronchitis, without the requirement for COPD to be diagnosed. More recently, a study in 1006 patients with moderate-to-severe COPD in China reported that long-term use of high-dose N-acetylcysteine (600 mg b.i.d.) was associated with a significant decrease in exacerbations compared with placebo (risk ratio 0.78; 95 % CI 0.67, 0.90; *p* = 0.0011) [113]. A smaller study in China has also confirmed a benefit of high-dose N-acetylcysteine (600 mg b.i.d.) for reducing exacerbations in high-risk patients [114]. Treatment with mucolytics appears to be well tolerated, with similar frequencies of adverse events compared with placebo [112, 113]. Erdosteine, a mucolytic agent with anti-inflammatory, antioxidant and bacterial anti-adhesive properties, has recently been reported to reduce the rate (17 %) and duration (44 %) of exacerbations compared with placebo in patients with COPD GOLD stage II-III and at least two exacerbations requiring medical intervention in the previous year [115].

In summary, while data indicate that the risk of exacerbation is reduced with mucolytic therapies compared with placebo in patients with COPD, as demonstrated in an updated systematic review, much heterogeneity exists meaning that current data should be interpreted cautiously [112].

#### Future therapeutic strategies

A multiple treatment comparison meta-analysis of 26 studies has compared the effects of various combinations of treatments and noted that, although combination therapies differ in their effects for reducing the risk of COPD exacerbations, they reduce risks more than monotherapy [116]. Further studies are required to confirm the optimal combinations for reducing exacerbations. However, randomized controlled studies to evaluate the effects of treatments on exacerbation risk are ongoing or have recently completed, such as the FLAME study comparing IND/GLY with SFC [74], and a comparison of umeclidinium/vilanterol/fluticasone furoate with fixed-dose dual combinations of fluticasone furoate/vilanterol and umeclidinium/vilanterol [NCT02164513] [117]. Various other combination therapies are being developed for the prevention of exacerbations, including triple therapies of ICS/LABA/LAMA [11]. In addition, combined PDE-3 and PDE-4 inhibitors (RPL554), monoclonal antibodies and p38 mitogen activated protein kinase inhibitors are in development for the prevention of exacerbations [11]. Benralizumab, an anti-interleukin-5 (IL-5) receptor alpha monoclonal antibody, has been investigated in Phase II studies in patients with sputum eosinophilia and COPD [118]. Although no significant benefit was noted with benralizumab on the rate of exacerbations in the overall study population, a non-significant numerical improvement was seen in subgroups of patients with elevated blood eosinophils [118]. Further studies with anti-IL-5 therapies are warranted.

## Conclusions

There are different risk factors and triggers for COPD exacerbations, and it is increasingly recognized that there are different phenotypes of patients who are at risk of exacerbations and who may vary in their response to treatments. Various approaches are available for reducing the risk of exacerbation, and identification of patient phenotypes may help treatments to be individualized to those who are most likely to benefit.

**Fig. 1:**
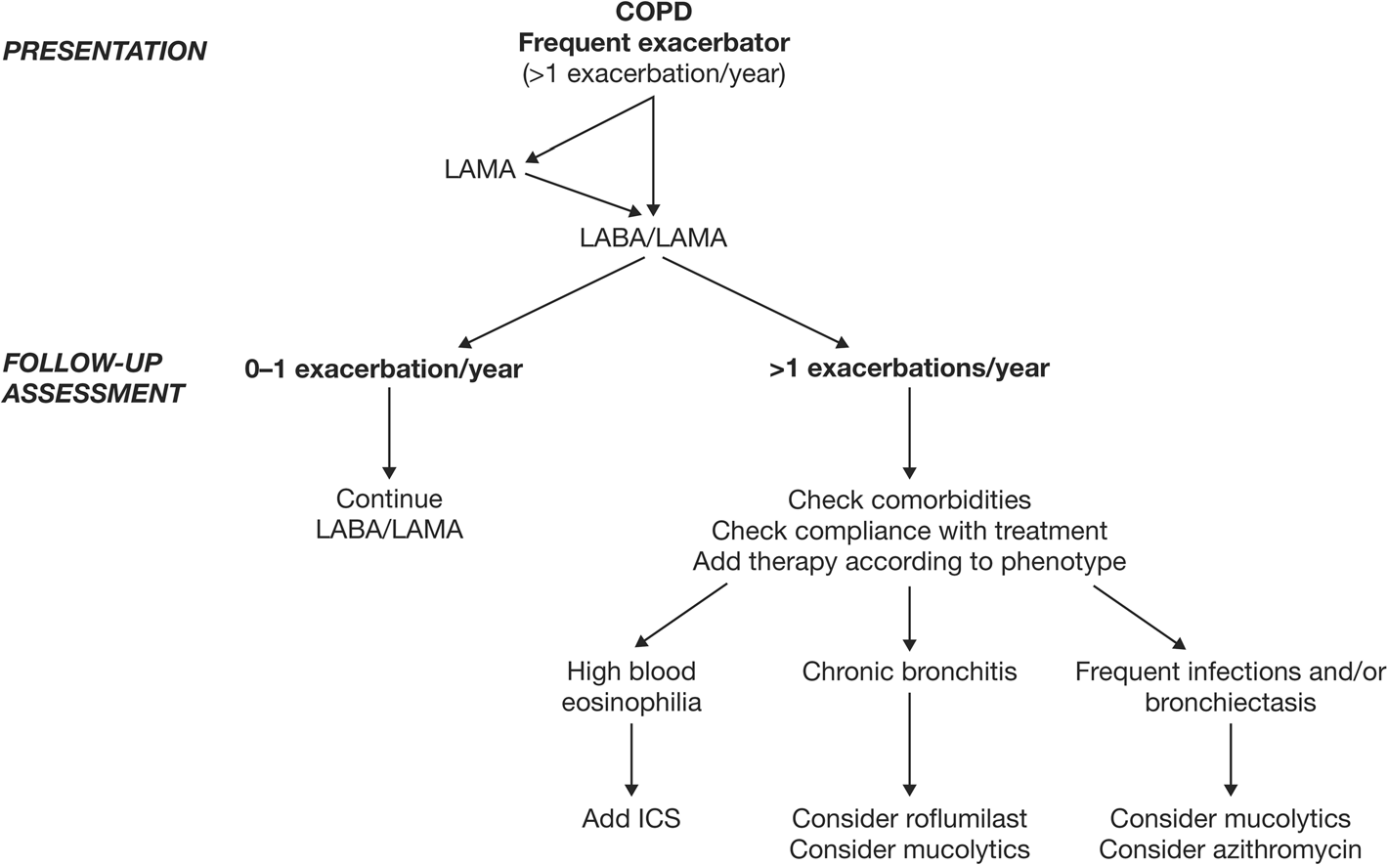
Therapeutic recommendations based on exacerbation phenotype. ICS, inhaled corticosteroid; LABA, long-acting β2-agonist; LAMA, long-acting muscarinic antagonist

Based on the available evidence reviewed herein, we believe that there is a strong case for maximizing bronchodilation as an initial strategy to reduce exacerbation risk in patients with COPD. For those who persist with exacerbations despite maximal bronchodilation, we advocate treatment according to their phenotype (Fig. 1) [119–121]. While current global guidelines recommend ICS treatment in symptomatic patients at high risk of an exacerbation (GOLD D), it should be borne in mind that this is based on a limited number of studies. Further, attempting to enhance the effect of ICS with additional theophylline does not reduce exacerbation rates [120]. Our evolving understanding in this area suggests that a more careful targeting of ICS may optimize the benefit-to-risk ratio. There is some evidence that ICS treatment is most effective in patients with higher eosinophil counts [95, 96], although these data are primarily from retrospective analyses and require further validation by prospective data. However, the recent FLAME study, which prospectively examined treatment effects according to baseline blood eosinophil count, found that the rate of exacerbations was significantly lower with IND/GLY compared with SFC irrespective of eosinophil count (i.e. <2 % or ≥2 %) [74]. Whether the outcome in FLAME was due to the fact that this was a comparison of a LABA/LAMA versus SFC, compared with other studies which have looked at a LABA compared with SFC, remains to be determined. Post hoc analysis of WISDOM suggests that withdrawal of ICS in the subgroup of patients with higher eosinophils may increase risk of exacerbation, but this also remains to be prospectively studied [121]. In exacerbators with chronic bronchitis we propose treatment with ICS (as per the Spanish GesEPOC guidelines [33]) or roflumilast, and in selected patient groups in whom ICS cannot be used, high-dose mucolytics. In exacerbators with chronic bronchitis who produce dark sputum and have previously required multiple courses of antibiotics, caution is advised regarding the use of ICS due to the increased risk of bacterial complications. A pragmatic approach may therefore be to use macrolides in reference centers with close clinical and microbiological follow-up. Finally, in exacerbators with emphysema we should emphasize the importance of maximal bronchodilation and consider adding ICS in patients with higher blood eosinophil counts. Additionally, non-pharmacologic therapy including pulmonary rehabilitation should be included as part of a comprehensive management plan for all patients at risk of exacerbation episodes due to benefits in terms of reductions in risk of hospitalizations.

## Additional file

Additional file 1: Supplementary material. **Table S1.** Comparison of guidelines and recommendations definitions of exacerbations of COPD. **Table S2.** Comparison of guidelines and recommendations assessment of exacerbation risk. (DOCX 30 kb)

## Abbreviations

ACOS: Asthma-COPD overlap syndrome
ATS: American Thoracic Society
COPD: Chronic obstructive pulmonary disease
ECLIPSE: Evaluation of COPD Longitudinally to Identify Predictive Surrogate Endpoints
ERS: European Respiratory Society
FEV1: Forced expiratory volume in 1 second
GOLD: Global initiative chronic Obstructive Lung Disease
ICS: Inhaled corticosteroid
LABA: Long-acting β_2_ agonist
LAMA: Long-acting muscarinic antagonist
PDE: Phosophodiesterase
REACT: Roflumilast and Exacerbations in patients receiving Appropriate Combination Therapy
UPLIFT: Understanding Potential Long-term Impacts on Function with Tiotropium

## Footnotes

1 Included in both systematic reviews

2 Included in the network meta-analysis.
