# Supplementary material for: Pharmacological strategies to reduce exacerbation risk in COPD: a narrative review
Source: Respir Res. 2016 Sep 10;17(1):112. doi: 10.1186/s12931-016-0425-5 (PMC5018159; doi:10.1186/s12931-016-0425-5)
Supplement: Supplementary file 1 — Supplementary material. Table S1. Comparison of guidelines and recommendations definitions of exacerbations of COPD. Table S2. Comparison of guidelines and recommendations assessment of exacerbation risk. (DOCX 30 kb) [file 12931_2016_425_MOESM1_ESM.docx]

**SUPPLEMENTARY MATERIAL**

**Supplementary Table 1.** Comparison of guidelines and recommendations definitions of exacerbations of COPD

| **Society** | **Definition of exacerbation** |
| --- | --- |
|  |  |
| GOLD strategy [1] | *An acute event characterized by a worsening of the patient’s respiratory symptoms that is beyond normal day-to-day variations and leads to a change in medication* |
| Spanish (GesEPOC)  [2, 3] | *An acute episode of clinical instability that occurs in the natural course of the disease, characterized by sustained worsening of respiratory symptoms beyond daily variations** |
| CPSS [4] | *Attacks of acute deterioration, which exceed the regular day-to-day symptom variability. These attacks last >2 days and require antibiotic treatment and/or systemic corticosteroids* |
| Saudi Thoracic Society [5] | *Acute sustained deterioration of respiratory symptoms in a COPD patient beyond day-to-day variability which could not be explained by any other cause, and this event may/may not lead to change in therapy* |
| ICS & NCCP [6] | *An acute event characterized*  *by sustained worsening of any of the patient’s respiratory symptoms (cough, sputum quantity and/or character, dyspnea) that is beyond normal day-to-day variation and leads to a change in medication, and where other causes of acute breathlessness have been clinically excluded* |

CPSS, Czech Pneumological and Phthisiological Society; GesEPOC, Guía Espanola de la EPOC (COPD); ICS, Indian Chest Society; NCCP, National College of Chest Physicians; GOLD, Global initiative for chronic Obstructive Lung Disease.

**Supplementary Table 2**  Comparison of guidelines and recommendations assessment of exacerbation risk

| **Society** | **Risk of exacerbation indicated by:** | |
| --- | --- | --- |
|  | **History of exacerbations** | **FEV_1_ / other** |
| GOLD strategy [1] | ≥2 in previous year *or* ≥1 leading to hospitalization in previous year | GOLD spirometric level 3 (severe) or 4 (very severe): FEV_1_ <50% predicted |
| Spanish (GesEPOC) [2, 3] | ≥2 in previous year | BODE/BODEx index to assess severity of disease |
| CPSS [4] | ≥2 exacerbations/year | Post-BD FEV_1_ 3 or 4 (as per GOLD) |
| Saudi Thoracic Society [5] | ≥2 exacerbations in past year *or* history of hospitalization due to COPD in past year (high exacerbation risk) |  |
| Finnish Medical Society Duodecim [7] | ≥2 exacerbations/year *or* ≥1 hospitalizations  due to COPD | Severe or very severe airflow limitation (FEV_1_ <50% predicted) |
| ICS & NCCP [6] | ≥2 exacerbations in last year | FEV_1_ <50% predicted |

BD, bronchodilator; BODE, Body mass index, airflow Obstruction, Dyspnea and Exercise capacity; COPD, chronic obstructive pulmonary disease; CPSS, Czech Pneumological and Phthisiological Society; FEV_1_, forced expiratory volume in 1 second; GesEPOC, Guía Espanola de la EPOC (COPD); ICS, Indian Chest Society; NCCP, National College of Chest Physicians; GOLD, Global initiative chronic Obstructive Lung Disease

**References**

1. Global Initiative for Chronic Obstructive Lung Disease (GOLD): **Global strategy for the diagnosis, management, and prevention of chronic obstructive pulmonary disease. Updated 2016. Last accessed 05 February 2016.** In *http://goldcopdorg/global-strategy-diagnosis-management-prevention-copd-2016/*; 2016.

2. Miravitlles M, Soler-Cataluña JJ, Calle M, Molina J, Almagro P, Quintano JA, Riesco JA, Trigueros JA, Piñera P, Simón A, et al: **Spanish guideline for COPD (GesEPOC). Update 2014.** *Arch Bronconeumol* 2014, **50 Suppl 1:**1-16.

3. Soler Cataluña JJ, Piñer Salmerón P, Trigueros JA, Calle M, Almagro P, Molina J, Quintano JA, Riesco JA, Simón A, Soriano JB, et al: **Spanish COPD guidelines (GesEPOC): hospital diagnosis and treatment of COPD exacerbation.** *Emergencias* 2013, **25:**301-317.

4. Koblizek V, Chlumsky J, Zindr V, Neumannova K, Zatloukal J, Zak J, Sedlak V, Kocianova J, Zatloukal J, Hejduk K, Pracharova S: **Chronic Obstructive Pulmonary Disease: official diagnosis and treatment guidelines of the Czech Pneumological and Phthisiological Society; a novel phenotypic approach to COPD with patient-oriented care.** *Biomed Pap Med Fac Univ Palacky Olomouc Czech Repub* 2013, **157:**189-201.

5. Khan JH, Lababidi HM, Al-Moamary MS, Zeitouni MO, Al-Jahdali HH, Al-Amoudi OS, Wali SO, Idrees MM, Al-Shimemri AA, Al Ghobain MO, et al: **The Saudi Guidelines for the Diagnosis and Management of COPD.** *Ann Thorac Med* 2014, **9:**55-76.

6. Gupta D, Agarwal R, Aggarwal AN, Maturu VN, Dhooria S, Prasad KT, Sehgal IS, Yenge LB, Jindal A, Singh N, et al: **Guidelines for diagnosis and management of chronic obstructive pulmonary disease: Joint ICS/NCCP (I) recommendations.** *Lung India* 2013, **30:**228-267.

7. Kankaanranta H, Harju T, Kilpelainen M, Mazur W, Lehto JT, Katajisto M, Peisa T, Meinander T, Lehtimäki L: **Diagnosis and Pharmacotherapy of Stable Chronic Obstructive Pulmonary Disease: The Finnish Guidelines.** *Basic Clin Pharmacol Toxicol* 2015, **116:**291-307.

**Searches for Tables**

**For tables 2–4, a pubmed search was conducted with the following search terms**

**LABA:** (indacaterol OR salmeterol OR formoterol OR olodaterol) AND COPD AND exacerbation

**LAMA:** (tiotropium OR umeclidinium OR glycopyrronium OR aclidinium) AND COPD AND exacerbation

**Inhaled corticosteroids:** (inhaled corticosteroid OR ICS OR fluticasone propionate OR fluticasone furoate OR budesonide OR beclomethasone) AND COPD AND exacerbation

**Mucolytics**: (mucolytics OR Carbocysteine OR N-acetylcysteine) AND COPD AND exacerbation

**PDE4 inhibitors**: (PDE-4 OR roflumilast) AND COPD AND exacerbation

**Antibiotics:** (Erythromycin OR Azithromycin OR macrolide antibiotics) AND COPD AND exacerbation

All searches were limited to clinical trial, English language and trials including >= 100 patients.

Search results were reviewed for relevance and to remove secondary analyses. Additional studies were included at the authors discretion based on knowledge of existing exacerbation data that may have been missed by the search.
